# *In silico *approaches to study mass and energy flows in microbial consortia: a syntrophic case study

**DOI:** 10.1186/1752-0509-3-114

**Published:** 2009-12-10

**Authors:** Reed Taffs, John E Aston, Kristen Brileya, Zackary Jay, Christian G Klatt, Shawn McGlynn, Natasha Mallette, Scott Montross, Robin Gerlach, William P Inskeep, David M Ward, Ross P Carlson

**Affiliations:** 1 Thermal Biology Institute, Montana State University, Bozeman, MT 59717, USA; 2 Center for Biofilm Engineering, Montana State University, Bozeman, MT 59717, USA

## Abstract

**Background:**

Three methods were developed for the application of stoichiometry-based network analysis approaches including elementary mode analysis to the study of mass and energy flows in microbial communities. Each has distinct advantages and disadvantages suitable for analyzing systems with different degrees of complexity and *a priori *knowledge. These approaches were tested and compared using data from the thermophilic, phototrophic mat communities from Octopus and Mushroom Springs in Yellowstone National Park (USA). The models were based on three distinct microbial guilds: oxygenic phototrophs, filamentous anoxygenic phototrophs, and sulfate-reducing bacteria. Two phases, day and night, were modeled to account for differences in the sources of mass and energy and the routes available for their exchange.

**Results:**

The *in silico *models were used to explore fundamental questions in ecology including the prediction of and explanation for measured relative abundances of primary producers in the mat, theoretical tradeoffs between overall productivity and the generation of toxic by-products, and the relative robustness of various guild interactions.

**Conclusion:**

The three modeling approaches represent a flexible toolbox for creating cellular metabolic networks to study microbial communities on scales ranging from cells to ecosystems. A comparison of the three methods highlights considerations for selecting the one most appropriate for a given microbial system. For instance, communities represented only by metagenomic data can be modeled using the pooled method which analyzes a community's total metabolic potential without attempting to partition enzymes to different organisms. Systems with extensive *a priori *information on microbial guilds can be represented using the compartmentalized technique, employing distinct control volumes to separate guild-appropriate enzymes and metabolites. If the complexity of a compartmentalized network creates an unacceptable computational burden, the nested analysis approach permits greater scalability at the cost of more user intervention through multiple rounds of pathway analysis.

## Background

Complex microbial communities drive the Earth's biogeochemical cycles [1]. In spite of their importance, the biochemical interactions within these communities are not yet well understood, nor have *in silico *methodologies for studying them matured. An improved understanding of how natural microbial communities mediate biogeochemical cycles will augment predictions of how these processes respond to disturbances from climate change to anthropogenic chemical deposition. Improved understanding may also provide a rational basis for using microbial consortia to produce biofuels and biomaterials from renewable resources [2].

Microbial communities described in terms of their environmental chemistry, ecophysiology, and phylogenetic diversity can be used as a foundation to develop, test, and compare *in silico *tools for analyzing community interactions. The phototrophic microbial mats of Octopus and Mushroom Springs of Yellowstone National Park (Wyoming, USA) represent an ideal test case due to the extensive available data [*e.g*. [3-9], and numerous other references found throughout this study]. These alkaline siliceous hot spring mats (50-74°C) are inhabited predominantly by unicellular cyanobacteria related to *Synechococcus *spp. and filamentous anoxygenic phototrophs (FAP) related to *Chloroflexus *and *Roseiflexus *spp. The community also contains sulfate-reducing bacteria (SRB) and other prokaryotes sustained by the primary productivity of the photosynthetic bacteria [3].

**Figure 1:**
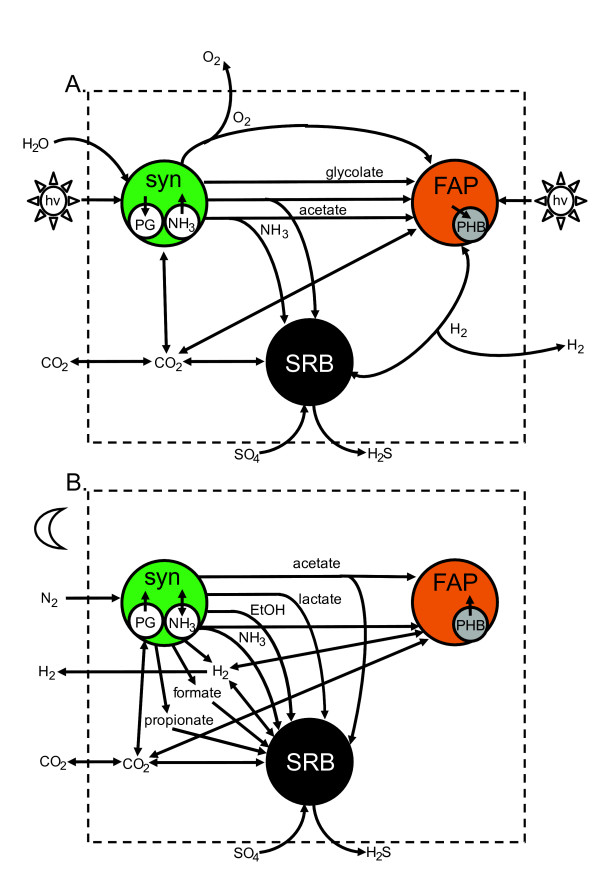
**Guild interactions**. Modeled consortium interactions are shown for (**A**) daylight and (**B**) nighttime simulations. Abbreviations: EtOH, ethanol; hv, photosynthetically-available photons; PG, polyglucose; PHB, polyhydroxybutyrate; syn, cyanobacteria (*i.e*. *Synechococcus*).

Previous studies of the mat have revealed diel (day-night) metabolic variation in various community members, driving shifts in the concentration and fate of dissolved metabolites [5,6,10-12]. Organic metabolites including glycolate and other acids, along with hydrogen, form the basis for mass and energy exchanges between community members (Figure 1A). For example, during the day, the photosynthetic cyanobacteria consume CO_2 _and produce O_2 _as a by-product of photosynthesis. High levels of O_2 _relative to CO_2 _promote oxygen competition at the ribulose-1,5-bisphosphate carboxylase/oxygenase (rubisco) active site, leading to the production of glycolate [9,13,14]. Other community members, including the photoheterotrophic FAP, can use glycolate as a carbon and energy source [15]. The cyanobacteria can also store excess photosynthate as polyglucose [16]. This carbon and energy storage material is fermented at night to organic acids (Figure 1B) [10]. FAP can incorporate fermentation products photoheterotrophically [12,15,17], while SRB appear to oxidize some of these products under anaerobic conditions [6,15,18].

Molecular level metabolic models were constructed to represent the central metabolism of three functional guilds thought to be important to material and energy flows through this community [12]. A guild is a group of species that exploit the same class of environmental resources in a similar manner [19]. Oxygenic photoautotrophs related to *Synechococcus *spp. were selected to represent the mat's primary source of fixed carbon and nitrogen. FAP from the family *Chloroflexaceae*, an important structural component of the mat community, were incorporated as metabolically versatile (photo)heterotrophs, capable of capturing light energy as phosphodiester bonds (cyclic photophosphorylation), but requiring externally supplied reducing equivalents. An SRB guild was included as a consumer of the metabolites produced by photosynthetic guilds. Other sub-dominant organisms (*e.g*. aerobic heterotrophic bacteria and methanogenic archaea) present in the mat [3] were not included in this first *in silico *representation of the system (see methods).

Metabolic networks were studied using the stoichiometry-based network analysis approach known as elementary mode analysis (EMA) [20-22]. EMA evaluates a metabolic network by decomposing the system into a complete set of the simplest biochemical pathways satisfying steady state conservation constraints with enzymatic fluxes occurring in physiologically reasonable directions (*i.e*. unidirectional enzyme-catalyzed reactions are not permitted to run backwards). These explicitly defined pathways are known as elementary modes and are frequently represented as vectors containing relative rates for every reaction in the metabolic network. The approach permits the description of any sustainable physiological behavior as a linear combination of the simplest biologically meaningful metabolic pathways [23,24].

Metabolic network modeling methodologies have been used extensively to analyze single organisms and monoculture systems [*e.g*. [25,26]]. An alternative stoichiometry-based technique, flux balance analysis, has been applied to a two-species laboratory microbial system [27]. That study used a compartmentalized approach analogous to strategies for modeling metabolic fluxes between organelles in eukaryotic organisms [28,29]. Topological approaches to metabolic network analysis have also been used at tree of life scales to analyze evolution of metabolism and the interactions of metabolic networks among species and with the environment [30-34]. However, these solely topological studies implicitly assume all genome encoded genes are simultaneously active with no consideration of critical reaction quantitative aspects like carbon and electron conservation relationships.

In the present study, novel methods for handling multispecies EMA are developed and applied to a microbial consortium representing a well-studied thermophilic community from Yellowstone National Park (YNP). To the best knowledge of the authors, this is the first time a stoichiometry-based network analysis approach has been applied to a natural, *in situ *microbial community and the first time these approaches have been used to analyze microbial biofilms/mats. Mass and energy fluxes were investigated using three different approaches each representing different levels of detail and computational complexity. The three approaches provide a general set of tools for studying microbial communities that can be selected based on the levels of available knowledge and community complexity. Each approach has advantages and disadvantages which are discussed along with examples illustrating how these methods can be used to gain insight into basic community metabolic processes. In particular, our work demonstrates a trade-off between knowledge of the distribution of metabolic capabilities between guilds in a consortium and the ability to conservatively predict and interpret limits for that community's behavior.

## Results

### Development and implementation of three consortium analysis approaches

The primary aim of this theoretical study was to develop, analyze, and compare different *in silico *approaches for molecular-level analyses of mass and energy flux through a microbial community. The following section details three distinct approaches, using the Yellowstone thermophilic phototrophic mat system as a test case. Advantages and disadvantages of each technique are highlighted and discussions detailing which approach is most appropriate for a given set of microbial data are provided. For each of the three methodologies discussed, both day and night scenarios were considered (Figure 1). All metabolic models, including an explicit listing of the reactions and metabolites, can be found in the additional file 1: Supplement.

### Compartmentalized consortium analysis approach

**Figure 2:**
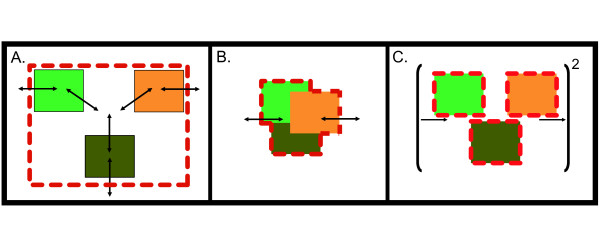
**Multi-species modeling approaches**. Conceptual depiction of the (**A**) compartmentalized, (**B**) pooled, and (**C**) nested consortium modeling approaches. Dashed red lines indicate system boundaries while thin solid lines represent guild boundaries.

A compartmentalized model was constructed in which each of the three guilds was a distinct compartment and exchangeable metabolites were transferred through a fourth compartment representing the extracellular environment (Figure 2A). This strategy has been used in the past for spatially and chemically segregated eukaryotic and microbial systems [27-29]. The approach was implemented by assigning reactions and metabolites to a network representing each guild (suffixes on metabolite identifiers prevented sharing of compounds common to the metabolism of multiple guilds), while explicit transport reactions accounted for the exchange of metabolites between guild members and the extracellular space. A short illustration of the separation mechanism is provided in additional file 1: Supplement.

The compartmentalized modeling approach has the advantage of conceptual tractability. Dividing the community into guild-level compartments linked by transferred metabolites (*e.g*. glycolate) is an intuitive way to represent interactions within a consortium. It is also an ideal method for understanding which guild performs a particular metabolic transformation. For example, the fraction of total biomass (carbon moles) or total maintenance ATP (used to account for energy-dependent cellular processes other than growth) produced by each guild is clear from the output.

One drawback to the compartmentalized approach is the sheer quantity of unique interactions possible. The size of the resulting network can lead to a 'combinatorial explosion' of elementary modes [35]. To address this limitation, the models for each guild member were constructed to capture the necessary metabolic capabilities while maintaining computational tractability (see methods section). A second drawback of the compartmentalized method is the requirement for significant *a priori *information or assumptions, as enzymes must be assigned to each separate guild. The Octopus and Mushroom Spring phototrophic mats are well-studied, permitting the use of compartmentalized results as a benchmark for the other two methodologies.

### Pooled reactions consortium analysis approach

The second approach, referred to as the pooled reactions consortium analysis, treated the consortium as a single entity (Figure 2B). All metabolic reactions and metabolites from the three guilds were combined into a single compartment. Reactions catalyzed by more than one guild were only considered once. The method captures the metabolic constraints of the overall matter and energy transformations without the need for detailed knowledge of every organism in the community.

The pooled reactions approach is ideally suited for investigating the metabolic potential of a community based solely on metagenomic data because the assignment of each reaction to a constituent guild is unnecessary. The technique is quite flexible and can be scaled to different levels of detail. An additional advantage of the pooled reactions approach is the reduction of computational burden. With these advantages, the method is uniquely suited for initial and exploratory analyses of diverse or poorly understood communities. For scientists accustomed to well-documented laboratory microbes, this approach may seem coarse however, the vast majority of organisms on the planet have not been cultured in the laboratory, much less isolated to purity [36]. This method represents a flexible starting point for analyzing such systems.

A weakness in the pooled reactions approach is that model output does not specify which guilds employ a particular enzyme or produce biomass and maintenance ATP. Instead, the results describe potential performance of the entire consortium. The method also neglects the logistics associated with transferring metabolites between organisms, including conversion of the given metabolite into one for which transporters are available. The approach is presented here using the well characterized cyanobacterial mats to highlight strengths and drawbacks to the method.

### Nested pathway consortium analysis approach

The third approach, termed the nested consortium analysis or EMA 'squared', uses successive rounds of EMA to analyze potential interactions within a consortium (Figure 2C). The first round of EMA is applied to each guild model in isolation. The output data are mined for ecologically relevant elementary modes, such as efficient biomass production from sunlight or efficient ATP generation from lactate and sulfate. The selected elementary modes are then compiled and used as input reactions for a second round of EMA to examine the potential for interactions between guilds. Conceptually, the first round of EMA provides guild-level stoichiometries relating substrates to products. These stoichiometries can be further processed in the same way that traditional EMA uses enzymatic stoichiometries to consider inter- and intraguild interactions. An example of the process is provided in additional file 1: Supplement.

The first round model output was explored for metabolic strategies representing 'selfish' and 'altruistic' operation of each guild. Selfish selection criteria were based on the efficient production of biomass and maintenance ATP. Altruistic selection criteria were based on efficient production of metabolites that could be consumed by other guilds, including for instance H_2_, NH_3_, acetate, and glycolate. Efficiency was evaluated by normalizing the amount of production by the required input (*e.g*. amount of glycolate or sunlight). A list of the ecological criteria considered can be found in additional file 1: Supplement.

Stable numerical implementation of the nested approach using CellNetAnalyzer v 9.0 required some heuristics. Elementary modes from the first round of analysis were normalized to vectors of unit length prior to the second round of EMA; without this step, errors occurred during calculation of elementary modes; specifically, the attempted inversion of matrices that are very nearly singular. Normalized coefficients were rounded to two decimal places to prevent similar problems. When rounding converted an otherwise non-zero coefficient to zero, the entire elementary mode was scaled so the smallest coefficient was equal to 0.01. The MATLAB script used for normalizing and rounding intermediate results is provided in additional file 1: Supplement. This step was necessary although it introduced a modicum of error. Error analysis is included in the following sections. These steps are not necessary if EMA is performed using the new bit pattern tree algorithm which is numerically more stable [37].

The nested pathway approach allows for the analysis of more complex metabolic networks and larger communities than the compartmentalized approach because each guild is initially considered separately. It also has the advantage of retaining guild-specific output lost in the pooled technique. The manual selection of specific modes permits a guided community analysis based on ecological strategies of interest for each guild; results can then be re-examined in light of the selection criteria used, relating community stoichiometry and guild strategy. The ability to assemble multiple sets of first-round output and concatenate the results allows the system to expand freely. The nested approach is unrelated to the decomposition approach for the analysis of large cellular networks with EMA [38], but is well-suited for reconnecting the output of the resulting subnetworks tractably.

The nested pathway analysis approach has the disadvantage of requiring two rounds of processing. In addition, manual selection of ecologically interesting modes from individual models requires *a priori *knowledge and can significantly influence the solution space. Finally, intermediate processing introduces some rounding error.

### Comparison of consortium analysis approaches

Two test cases were analyzed to compare the three approaches in terms of their ability to describe and explain the flows of carbon, nitrogen, and energy through the mat community. The first case study considered daylight production of biomass fueled by solar energy, while the second case study considered the fermentation of stored polyglucose to drive nitrogen fixation during the night. Table 1 highlights key model outputs associated with the different daytime simulations, including the number of modes identified as well as important parameters like maximum yield of biomass or maintenance ATP per photosynthetically-available photon. Table 2 shows similar results for the nighttime simulations.

**Table 1:** Elementary mode analysis output summary for comparison of daylight modeling approaches.

	compartment	pooled	nested
# of modes	74507	38216	428
Y_X/hv_^a^	0.117	0.128	0.116
Y_ATP/hv_^b^	1	1	1
Y_Xsyn/hv_^c^	0.117	n/a	0.116
Y_XFAP/hv_	0.078	n/a	0.075
Y_XSRB/hv_	0.016	n/a	0.015
Y_ATPsyn/hv_	1	n/a	1
Y_ATPFAP/hv_	0.5	n/a	0.506
Y_ATPSRB/hv_	0.033	n/a	0.031
Y_PG/hv_	0.122	0.125	0.121
Y_PHB/hv_^d^	0.089	0.111	0.090

n/a = not applicable.

^a ^'Y_i/j_' is the yield of i on j (*i.e*. production of i divided by consumption of j). 'X' represents Cmoles of biomass and 'hv' denotes moles of photosynthetically-available photons.

^b ^'ATP' represents moles of ATP available for cellular maintenance.

^c ^'syn' designates a parameter associated with the *Synechococcus *guild. 'FAP' indicates a parameter associated with the filamentous anoxygenic phototroph guild and 'SRB' denotes a parameter associated with sulfate-reducing bacteria guild.

^d ^'PHB' represents Cmoles of polyhydroxybutyrate (a carbon/electron storage compound associated with the filamentous anoxygenic phototrophs).

**Table 2:** Elementary mode analysis output summary for comparison of nighttime modeling approaches.

	compartment	pooled	nested
# of modes	14004	36233	46038
Y_cypc/PG_^a^	0.063	0.103	0.063
Y_cypc/SO4_^b^	0.259	0.265	0.203
Y_X/PG_^c^	0.218	0.229	0.209
Y_X/SO4_	0.737	0.744	0.692
Y_ATP/PG_^d^	0.958	0.958	0.976
Y_ATP/SO4_	2.25	2.25	2.11
Y_Xsyn/PG_^e^	0.148	n/a	0.150
Y_XSRB/PG_	0.218	n/a	0.184
Y_ATPsyn/PG_	0.5	n/a	0.5
Y_ATPSRB/PG_	0.625	n/a	0.638

n/a = not applicable.

^a ^'Y_i/j_' is the yield of i on j (*i.e*. production of i divided by consumption of j). 'cypc' denotes moles of nitrogen fixed and stored as cyanophycin, while 'PG' represents Cmoles of polyglucose.

^b ^'SO_4_^' ^represents moles of sulfate used as an oxidant.

^c ^'X' represents Cmoles of biomass.

^d ^'ATP' represents moles of ATP available for cellular maintenance.

^e ^'syn' designates a parameter associated with the *Synechococcus *guild. 'FAP' indicates a parameter associated with the filamentous anoxygenic phototroph guild and 'SRB' denotes a parameter associated with sulfate-reducing bacteria guild.

### Case one: Consortium photo-efficiency

The relationship between solar energy input and community productivity is a fundamental subject of ecological inquiry, as is the relative abundance of various species. The model community is an example of how metabolic knowledge feeds into these questions, providing a natural point for comparison of the three methods. Oxygenic photosynthesis drives primary productivity in the community by splitting water into reducing equivalents and O_2_. The reducing equivalents can be used to fix CO_2 _via the Calvin-Benson-Bassham cycle. The O_2_, however, can compete with CO_2 _for the rubisco active site, resulting in the production of glycolate instead of an additional Cmole of reduced carbon (equations 1 and 2). A Cmole is a mole of carbon atoms; one mole of glucose (C_6_H_12_O_6_) is equivalent to six Cmoles of glucose.(1)

Experimental characterization of these fluxes from the Octopus and Mushroom Spring microbial mats has been reported. The O_2_/CO_2 _flux ratio at the rubisco active site is estimated to be approximately 0.03-0.07 when dissolved gas concentrations represent mid-afternoon conditions at the springs [9]. The derivation of these estimates from the reported data is demonstrated in additional file 1: Supplement.

**Figure 3:**
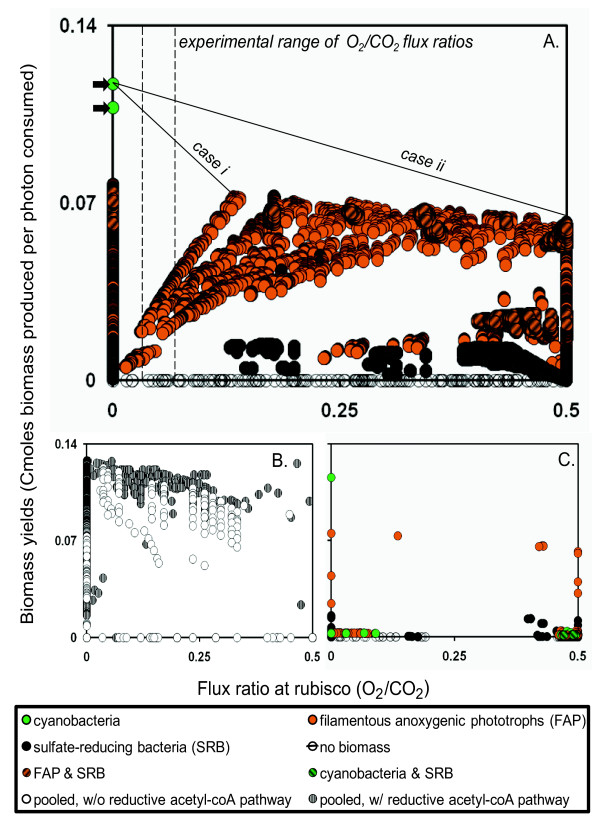
**Daytime case study output**. Daylight biomass-producing elementary modes, plotted for (**A**) compartmentalized, (**B**) pooled, and (**C**) nested simulations in terms of total consortium biomass yield on photons (Cmole/mole) and oxygen competition at the rubisco enzyme (O_2 _flux relative to CO_2 _flux). Each point represents a single mode. Solid line segments in (**A**) connect modes combined to represent ecological scenarios, while dashed lines represent the experimentally-derived limits for oxygen competition at the rubisco enzyme in the mid-afternoon. Arrows denote modes producing cyanobacterial biomass (see text for more details). Points are color-coded by biomass-producing guild (**A **&**C**) or SRB-associated reductive acetyl-CoA activity (**B**).

The output from the three *in silico *community analysis approaches was plotted using two physiological variables thought to be important to community interactions: 1) O_2 _competition at the rubisco enzyme (O_2_/CO_2_) and 2) the quantity of biomass (Cmole) synthesized per mole of absorbed photons (Figure 3). Relationships between these properties are examined below as a vehicle for comparison of the three modeling approaches.

### Compartmentalized consortium analysis

The compartmentalized approach, wherein guilds interact through a mass-balanced external compartment (see section 1a, Figure 2A, and additional file 1: Supplement), served as a basis for comparison of the community modeling techniques. This method revealed only two elementary modes (out of a total of 74,507 distinct elementary modes) producing cyanobacterial biomass. These modes, highlighted with arrows in Figure 3A, differ based on ATP production strategy. The higher-yielding elementary mode generated ATP by oxidizing reducing equivalents from non-cyclic photophosphorylation, while the lower-yielding elementary mode produced ATP through cyclic photophosphorylation.

All other biomass-producing elementary modes generated FAP, SRB, or a combination of FAP and SRB biomass. At low levels of O_2 _competition at the rubisco enzyme, Figure 3A illustrates that optimal FAP and SRB biomass synthesis efficiency increases with oxygen competition (positive trend for highest biomass-yielding elementary modes for each increment of oxygen competition). This is due to an increase in glycolate production, shuttling electrons either directly or indirectly to the other guilds. The trend continues until a rubisco O_2_/CO_2 _flux ratio near 0.13 is reached. At O_2 _competition levels greater than about 0.17, O_2 _interferes with the efficient photon-based production of cellular carbon (*i.e*. the reducing equivalents liberated with the oxygen are insufficient to cover the costs of processing the additional glycolate).

Theoretical ecological studies have suggested that organisms adapted to a biofilm or mat lifestyle will draw on available resources in a manner which maximizes yields and thus favors the whole community [39,40]. Combining this theory with the experimentally determined O_2_/CO_2 _competition at the rubisco enzyme made it possible to predict relative yields of the cyanobacteria and FAP. Two different cases were considered. The first case (*i*) examined the ramifications of each guild striving to maximize its own biomass yield and the second case (*ii*) examined different guilds working in concert to maximize biomass for the entire consortium (see Figure 3A).

Cellular metabolism can be expressed accurately as a linear combination of distinct elementary modes [24,41]. It is hypothesized that a consortium's metabolic activity can also be described using linear combinations of the elementary modes (with non-negative coefficients). When exactly two elementary modes are considered, the contribution of each mode to the overall metabolic process (represented by a point on the connecting line segment) is fixed by the ratio of any two fluxes for which the constituent modes have non-identical ratios. That relationship is demonstrated in additional file 1: Supplement. For each considered case, a combined metabolism was identified using the ratio of fluxes through rubisco (O_2_/CO_2_) derived from experimental data [9]. The derived values were used to bound the solution space (*i.e*. set of acceptable flux distributions).

This relationship was applied to the highest-yielding biomass-producing modes from the compartmentalized model to predict the relative abundance of cyanobacteria and FAP for a fixed photon input budget (line segments in Figure 3A connect the elementary modes of interest). Modes producing SRB biomass could easily be combined in the same way, but were not among the highest-yielding strategies. The scenario normalized biomass to a per photon basis. The case *i *scenario predicts ratios of cyanobacteria to FAP biomass production ranging from 2.5:1 to 6.5:1 (cyanobacteria to FAP) based on the experimentally determined oxygen competition at the rubisco enzyme. The case *ii *scenario predicts ratios from 8.2:1 to 17.7:1 on the same basis. The numerical procedure is demonstrated in additional file 1: Supplement. The analysis control volume consists of the top 1 mm of mat. The control volume growth is exactly equal to the rate at which biomass leaves the control volume. Physically speaking, this represents the new growth burying the old growth.

The ratio of cyanobacterial to FAP biovolume in a Mushroom Spring mat sample was experimentally measured to be 1.6:1 [16]. It is assumed here that biomass and biovolume are related in the same manner for both species. The relative abundances of metagenomic reads suggest biomass ratios for these guilds between about 1.5:1 and 3.5:1 in the top 1 mm of Octopus and Mushroom Spring mats, assuming no bias in the sampling and sequencing pipeline (Klatt *et al*., in preparation). The reported ratios are closer to the modeled scenario where each individual guild seeks to maximize its own biomass yields rather than the scenario optimizing biomass yield of the entire consortium. A potential explanation for the modest discrepancy between prediction and experimental data is the experimental underestimation of glycolate production due to consumption of radiolabeled photosynthate during incubation. Increasing the flux ratio (O_2_/CO_2_) at rubisco (to correspond with higher glycolate production rates) drives the solution space for the case *i *scenario into stronger agreement with experimental values much more quickly than for the case *ii *scenario. It should also be noted that *in situ *environmental systems vary considerably in both space and time, and the flux ratios were derived from measurements at a single condition.

### Pooled reactions consortium analysis

The same variables were plotted for elementary modes identified with the pooled technique, wherein all reactions and metabolites were combined into a single metabolic unit (Figure 3B). The predicted maximum community biomass efficiency was 9.4% higher for the pooled approach than the compartmentalized approach. The cyanobacteria (responsible for the highest-yielding, compartmentalized biomass mode) do not possess a full citric acid cycle; with the pooled treatment, however, the entire cycle becomes available, efficiently catabolizing photosynthate without the usual logistical costs of metabolite transport between guilds. The genome of RS-1, a FAP isolated from a hot spring microbial mat, appears to code for the enzyme missing from OS-A and OS-B', sequenced cyanobacteria from Octopus Spring.

Contrary to the compartmentalized approach, the pooled output suggests that oxygen competition at the rubisco active site does not necessarily affect the biomass yield per photon (note the nearly flat trend of uppermost points in Figure 3B). This represents an uncoupling of heterotrophy and photorespiration, neglecting the role of glycolate as a transfer metabolite. These seemingly contradictory elementary modes all utilized the SRB-associated reductive acetyl-CoA CO_2 _fixation pathway, and are striped gray in Figure 3B. This pathway is metabolically inexpensive in the pooled approach due to the direct availability of reducing equivalents derived from oxygenic photosynthesis. These enzymes are generally very sensitive to O_2 _[42], calling the feasibility of these particular elementary modes into question. SRB activity has been reported in the upper 0.5 cm of the mat during day [6], but the activity may be limited to small anoxic microenvironments and is unlikely to represent a major daytime CO_2 _fixation strategy.

Filtering out elementary modes using the reductive acetyl-CoA pathway leaves a trend of decreasing optimal biomass per photon yields with increasing O_2_/CO_2 _flux ratios at the rubisco enzyme. The trend appears very similar to case *ii *from the compartmentalized analysis as a result of the pooled approach's treatment of biomass. A non-guild-specific biomass term means that maximizing the yield optimizes production of biomass for the consortium as a whole, the same criteria used to generate the case *ii *scenario.

This analysis highlights potential challenges associated with the pooled community approach. First, pooled reactions can result in higher predicted yields than compartmentalized reactions because there are no logistical costs associated with inter-guild metabolite transfer. The second challenge is the combination of enzymatic activities that are not compatible. The use of oxygenic photosynthesis to drive extremely oxygen-sensitive enzymes, for instance, seems unreasonable. The pooled analysis generates more appropriate trends if *a priori *information regarding the oxygen sensitivity of the reductive acetyl-CoA enzymes is applied as a filter. In addition, because enzymatic activities are shared rather than apportioned between individual guilds, maximizing any yield does so for the entire consortium rather than a single guild. This approach is well suited for initial work on systems lacking sufficient *a priori *data to construct a compartmentalized model. The fact that such data is lacking for the vast majority of all natural ecosystems emphasizes the usefulness of this approach.

### Nested consortium analysis

The nested approach produces similar results to the compartmentalized method. While this may not be readily apparent from a cursory comparison of Figures 3A and 3C, connecting the extreme upper-left and upper-right points on each subfigure shows that the limits of the solution space (in terms of biomass yield on photons and oxygen competition at the rubisco enzyme) are quite comparable, although the nested approach identifies fewer suboptimal solutions. The biomass yield on photons decreases with increasing O_2_/CO_2 _flux ratios at the rubisco enzyme. The initial increasing trend seen in the compartmentalized model consisted of suboptimal solutions not identified as ecologically relevant in the first-round processing. Those metabolic behaviors are all outperformed by linear combinations denoted by the case *i *line in Figure 1A. The optimal biomass yield on photons is equivalent (within the error introduced by rounding) to the compartmentalized prediction. Combination of modes, as in the case *i *compartmentalized scenario, identifies very similar ratios of biomass productivity for cyanobacteria to FAP (from 2.0:1 to 5.9:1) at the O_2_/CO_2 _flux ratios of 0.03-0.07 suggested by experiment (calculations are demonstrated on the compartmentalized output in additional file 1: Supplement). The total number of elementary modes is lower than the compartmentalized approach (428 vs. 74,507) because the initial round of EMA selects only strategies deemed ecologically competitive or relevant. The first round of processing removes many mathematical solutions that are not necessarily of interest for additional analysis. Caution is required; neglect during first-round selection can prevent discovery of interactions or cause underestimations of metabolic potential.

Any system characterized well enough to construct a compartmentalized model can also be analyzed using the nested approach. The approach is best suited for complex systems that prove computationally difficult to analyze using a compartmentalized simulation. In addition, the nested community analysis approach facilitates interrogation of the competitive strategies employed by individual guilds to produce a compartmentalized modeling result. For instance, the experimentally relevant case *i *results shown in Figure 3A (biomass production efficiency optimized by guild rather than as a community) require the FAP guild to maximize biomass yield (based on carbon influx) for the strictly heterotrophic co-metabolism of glycolate and acetate. The cyanobacterial guild must concurrently operate a metabolism comprised of three distinct elementary modes: the highest yielding strategies for biomass, glycolate, and acetate production all based on photons. This information is embedded in the criteria used to select the first-round elementary modes.

The nested community analysis approach requires the elementary mode coefficients to be normalized and rounded. Error analysis was performed to gauge the effect of this processing on the results. Each mode was examined for the error in carbon fixed and the amount of absorbed energy. The maximum error introduced was 0.004 Cmoles per mole of photons. The complete error results from both case studies are included in additional file 1: Supplement.

### Case two: Nitrogen fixation

Nitrogen fixation fueled by the fermentation of polyglucose is thought to be an important process in the mat during night and early morning [5,11]. The presented models only consider nitrogen fixation at night, since the oxygen sensitivity of nitrogenase should render it inoperable during the day when the mat is superoxic. Future modeling efforts will consider the morning separately, acknowledging evidence that suggests a large fraction of nitrogen fixation *in situ *occurs after sunrise but before O_2 _production exceeds consumption [5].

The second case study examined the efficiency of N_2 _reduction with polyglucose and the corresponding production of five potentially inhibitory or toxic fermentation by-products: formate, acetate, ethanol, lactate, and propionate. Their toxicities have not been explicitly characterized in this system, but their total molar yield on polyglucose represents a convenient 'toxicity parameter' for comparison of modeling approaches. It should be noted, however, that nitrogen fixation and the incidental production and fate of fermentation products are separately questions of ecological interest.

The FAP guild was included in the night-time simulations. They were inactive however, because unlike the SRB, they are incapable of drawing on sulfate as an electron acceptor. While the FAP guild can jettison electrons as hydrogen, this requires energy input from light or an alternative oxidant (oxygen was assumed to be unavailable during the night scenario).

**Figure 4:**
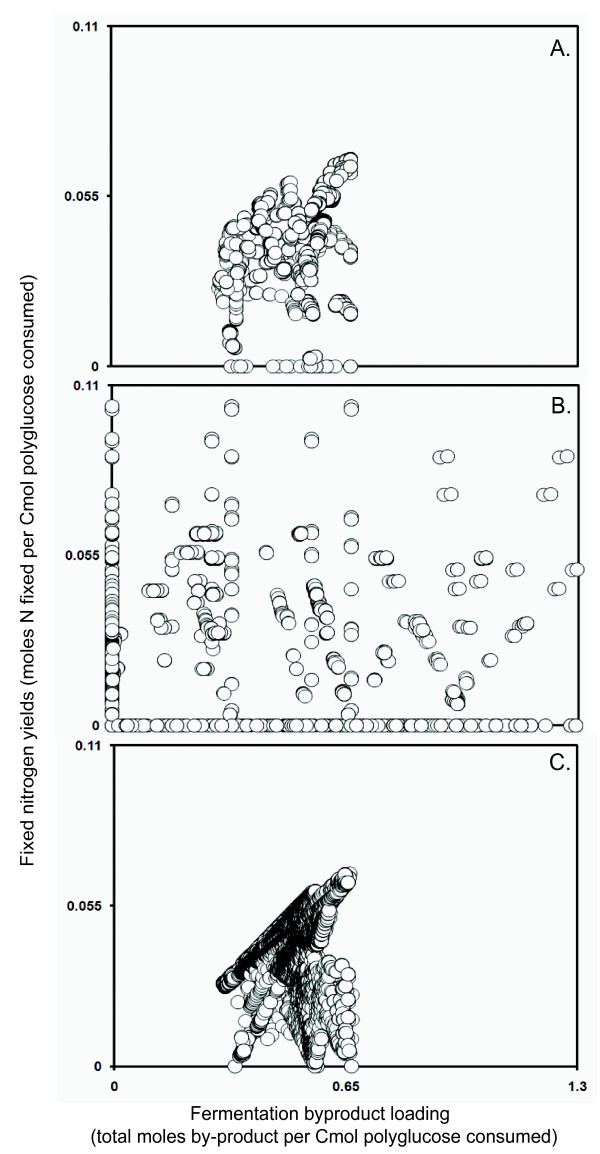
**Night case study output**. Nighttime nitrogen-fixing elementary modes, plotted for (**A**) compartmentalized, (**B**) pooled, and (**C**) nested consortium simulations in terms of total fixed nitrogen yields on polyglucose (Nmole/Cmole) and fermentation byproduct loading (total moles of fermentation by-products produced for each Cmole of polyglucose consumed). Each point represents a single mode.

Figure 4A depicts the relationship between the secretion of fermentation products and the efficiency of the metabolic strategy according to the compartmentalized results. The high density of points is an explicit illustration of the system robustness. As the yield of NH_3 _synthesis increases, more fermentation products are produced. If heterotrophic guilds such as the SRB rapidly metabolize the fermentation products (or those products are removed efficiently through physical transport processes), the high-yielding strategy would likely be the most ecologically competitive strategy for the cyanobacteria. If, alternatively, fermentation products are building up to toxic levels, than the mode associated with lowest by-product loading could be preferred.

The pooled results in Figure 4B demonstrate the effects of neglecting constraints on the exchange of metabolites between guilds. The results suggest, in contrast to predictions by the other two models, that nitrogen fixation at night is possible without associated production of potentially inhibitory or toxic fermentation products (all points in Figure 4B located on the y-axis above the origin). Similar to the photo-efficiency test case, the pooled analysis provides a less conservative solution space than the other approaches. Less information is required to build the pooled model, but predicted yields have to be viewed as theoretical upper limits, with the actual consortium probably operating at lower yields. The approach offers a rational, constraint-based approximation to the metabolic potential of a poorly characterized microbial consortium.

The night nested results have nitrogen-fixing efficiencies similar to the compartmentalized approach. The similarity of the shape and size of the solution spaces between nested and compartmentalized techniques (Figures 4A and 4C) indicates that modes of interest were selected appropriately during first-round processing. Considering the corresponding first-round criteria allows mapping of results into ecological strategies for individual guilds. For example, in the elementary mode resulting in the highest yield of fixed nitrogen on polyglucose, the cyanobacteria guild uses two strategies, maximizing production of free NH_3 _and cyanophycin and co-producing acetate, formate, and ethanol. Three distinct SRB physiologies are employed, maximizing biomass production on each fermentation product individually. Error analysis on the night nested model was performed by comparing error in the carbon balance with polyglucose consumption for every mode. The largest error due to rounding was a reasonable 0.033 Cmoles for every Cmole of polyglucose consumed. The complete error results from both case studies are included in additional file 1: Supplement.

### Consortium food web robustness analysis

**Figure 5:**
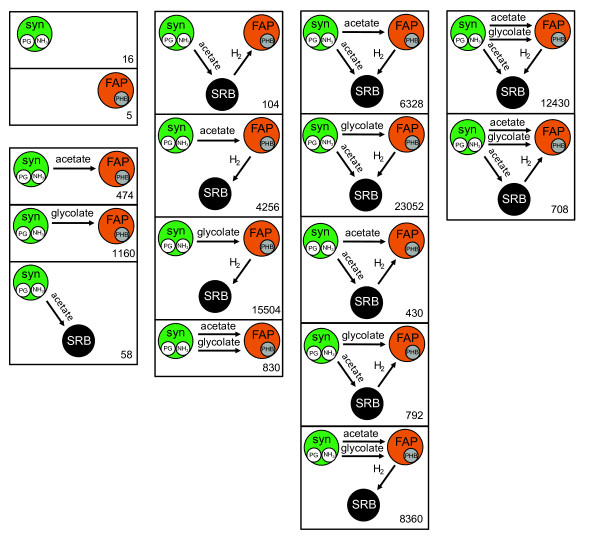
**Daytime compartmentalized guild interaction classes**. All 74,507 elementary modes identified in the daylight compartmentalized model, binned into 16 classes of guild interactions. Number of modes belonging to each classification is listed in lower right. Columns from left to right contain interaction classes requiring an increasing number of inter-guild metabolite transfers. Abbreviations: PG, polyglucose; PHB, polyhydroxybutyrate; syn, cyanobacteria (*i.e. Synechococcus*).

The compartmentalized consortium analysis approach was used to explore additional community properties. Metabolic networks within individual cells are highly branched, forming robust and decidedly redundant systems thought to be resilient to perturbations and disruptions [34,43-45]. Figure 5 details how the 74,507 unique daytime elementary modes can be divided into 16 different inter-guild mass and energy exchanges. Interactions associated with a large number of elementary modes are more likely to remain functional during environmental perturbations [46,47].

Figure 5 indicates that a large number of metabolic strategies (93.9% of elementary modes revealed by the compartmentalized simulation) exist for the daytime transfer of carbon and electrons from the cyanobacterial guild to the FAP guild with associated FAP production of H_2 _providing electrons to the SRB guild. In fact, daylight SRB activity *in silico *is overwhelmingly dependent on such hydrogen (97.1% of compartmentalized elementary modes with non-zero SRB fluxes). There are only 58 unique elementary modes involving the transfer of acetate from the cyanobacteria to SRB which do not involve any FAP activity. The interaction analysis also highlights the importance of the well-connected and functionally versatile FAP guild in the mat community metabolism. Only 74 out of the 74,507 unique elementary modes (0.1%) identified by the daytime compartmentalized simulation can function without the FAP guild.

## Discussion

Three *in silico *consortium analysis methods were developed and applied to a thermophilic phototrophic mat community from Yellowstone National Park as a test case. The results of this systems ecology study demonstrate the applicability of consortium EMA, explicitly mapping the genes-to-function emergent properties associated with the connectivity of metabolic reactions via the exchange of mass and energy. Since the presented techniques are culture-independent, they are compatible with metagenomic approaches and other studies in which culturing or isolation has proven difficult. This study provides a foundation for further work to refine and test the models using co-culture studies; potential inquiries include the measurement of substrate uptake and growth rates, biomass compositions, mRNA and protein expression, and quantification of internal fluxes. Toxicity profiles of the various fermentation products to representative microbes would also be of interest. Representative organisms or enrichment cultures are available for each guild, and the results of such experimentation will be quite useful for model validation and improvement. That work, however, is beyond the scope of the current study, which seeks only to provide methods for the extension of EMA to microbial consortia.

An alternative approach to the mass-balance-based analysis of large networks, flux balance analysis[48,49], has been applied to genome-scale metabolic networks[50], which is currently not possible with EMA [37]. Flux balance analysis has been used to investigate central carbon and energy metabolism of a two species consortium [27]. Flux balance analysis could likely be applied to the presented model system and could be used to ask similar questions. The output, however, would be a single flux distribution denoting an optimal use of the network as defined by an objective function, rather than a generating set for all flux distributions allowed by the network (although methods have been established to examine the effects of variations in external constraints on the value of the objective function[51]). As the choice of an appropriate objective function is not always clear prior to extensive experimentation [52,53], a generating set is useful for exploratory modeling. The utility of a generating set for explanatory modeling has also been recently demonstrated [24]. These benefits justify the development of methods for the application of EMA to multispecies systems alongside large-scale network methods. It is worth considering how the methods developed here will be affected by larger network sizes. This is important because potential applications include more diversified communities and more complex cellular metabolisms. Systems cast as compartmentalized models which prove to be too computationally heavy for current EMA algorithms can be analyzed using the nested approach.

The compartmentalized community analysis method has the advantage of intuitive tractability and separates activity and function by guild, but requires substantially more knowledge of the community than the pooled reactions approach. Tables 1 and 2 illustrate how the energy added to electrons during oxygenic photosynthesis is dissipated with the movement of mass and energy through the consortium. The cyanobacteria exhibit a predicted maximum biomass yield of 0.117 Cmoles for each mole of photons absorbed by the mat. This value is 0.078 for the FAP guild, with the difference providing a rough measure of the cost of transferring reducing equivalents between guilds. The SRB also rely on the by-products of the primary producer guilds for electrons, but are further constrained by an inability to derive energy from light or respiration on O_2_. The highest SRB biomass yield predicted by the daytime compartmentalized model is 0.016 Cmoles per absorbed mole of photons, slightly more than one tenth of the cyanobacterial yield. The ratio between theoretical optima for photosynthetic and SRB biomass yields is similar to observed ratios between primary producers and the secondary productivity of consumers in macroscale ecological systems [19,54].

The compartmentalized method also lends itself uniquely to investigation of the robustness of specific consortium interaction types (see Figure 5). Investigating interactions in the model showed that the FAP are central to the metabolic capabilities of the consortium as a whole; FAP involvement was required for the vast majority (99.9%) of elementary modes. Finally, the highest-yielding biomass-producing modes from the compartmentalized approach were combined to show that experimental data supports strategies optimizing productivity by individual guilds more strongly than strategies optimizing productivity for the entire consortium [[9,16]; Klatt et al., in preparation].

The pooled reactions consortium analysis method modeled community metabolic potential by treating all enzymatic activities and metabolites as residents of the same physical space, without membrane boundaries. The pooled reactions approach represents the coarsest-scale methodology, requires the least *a priori *information, and is easier to implement than alternative approaches. The pooled approach can often be used when other approaches cannot (due to complexity) or should not (due to lack of detailed data). These advantages are balanced against a tendency to overestimate the metabolic potential. This is unsurprising, as real communities are not super-organisms; individuals are membrane-separated and must contend with the logistics associated with matter and energy transport. The pooled technique is best for initial work on 'poorly' characterized systems. The vast majority of ecosystems are not characterized to a level permitting a compartmentalized analysis, suggesting the pooled approach will be relevant for a long time to come.

Finally, the nested community analysis has properties very similar to the compartmentalized approach, but with the important advantage of easy scalability, achieved by concatenating multiple rounds of EMA analysis. The approach also provides additional ecological insight into the competitive strategies underlying each guild's function. This information is contained within the criteria used to select first-round building blocks. The nested method also easily captures interactions between different guilds as well as between members of the same guild expressing different physiologies. For instance, different cyanobacterial elementary modes can combine, representing exchange of metabolites between cyanobacteria expressing different metabolic activity due to differing positions across spatial gradients of light, temperature, and concentration. This type of exchange appears to be very relevant to actual mat function, as other work suggests the presence of functionally distinct *Synechococcus *populations that are adapted to particular microenvironments [55]. As with the compartmentalized approach, the daylight case study found that optimal guild strategies were more strongly supported by experimental data than optimal consortium strategies.

Combinations of the pooled and compartmentalized methods can be used to provide information not easily obtainable with any single approach. As an ecologically relevant example, a cost-benefit relationship was quantified for a complete citric acid cycle in the cyanobacterial guild, as opposed to the incomplete cycle indicated by the genomic sequence of relevant isolates. *Synechococcus *spp. OS-A and OS-B' genomes do not contain the oxaloacetate-producing malate dehydrogenase. The pooled reactions modeling approach identified a cluster of optimal elementary modes using a primarily cyanobacterial metabolism along with the missing citric acid cycle enzyme, which is FAP-derived. The biomass yields in this cluster are a 9.4% improvement relative to purely cyanobacterial metabolic potential, given by the compartmentalized methodology. This is a large difference in yields and laboratory evolution experiments have shown that much smaller differences (0.5% difference in growth rate) are selectable under competitive conditions [56]. These hot spring mat communities are thought to be modern derivatives of ancient prokaryotic communities, and the long history of cyanobacterial and FAP cohabitation suggests the potential for horizontal gene transfer. Evidence for actual horizontal gene transfer events between cyanobacteria and FAP has been published elsewhere [57]. The cyanobacteria in our study have not acquired and passed on the missing Krebs cycle gene, suggesting that the costs of production, maintenance, and regulation for malate dehydrogenase are unlikely to be offset by the benefit of a 9.4% improvement in biomass yield.

## Conclusion

Each of the three *in silico *modeling approaches developed in this study provides a mathematical description of physical constraints on metabolic activity in a consortium. These techniques allow the extension of EMA to ecologically relevant multi-species, biofilm systems. Their contrasting strengths can be combined to arrive at a more holistic description than is possible with any of the methods alone, allowing a broad perspective from which to frame observations and base predictions. These approaches can be adapted to a wide range of microbial communities including both natural and anthropogenic systems. Potential applications include modeling communities involved in wastewater treatment, bioprocess engineering, and environmental remediation, as well as the study of host-pathogen interactions in medicine and symbiotic relationships such as nitrogen-fixing root communities.

## Methods

### Metabolic models

The metabolic models presented in this study exist in a control volume consisting of the upper 1 mm of the microbial mat. The steady state control volume considers biomass production but does not include an explicit biomass degradation term. To maintain steady state, biomass would leave the control volume at the same rate it is produced. This would correspond with the physical process of new growth burying old growth. Central carbon and energy metabolism network models for each guild considered to be in the control volume were constructed from literature reviews and annotated genomes of representative organisms including *Synechococcus *spp. OS-A and OS-B' [58] as well as *Roseiflexus *sp. RS-1 [59]. GenBank accession numbers are CP000686.1, CP000239.1, and CP000240.1 for the RS-1, OS-A, and OS-B' genomes, respectively. These genomes are highly representative (>90% nucleotide identity to metagenomic reads) of native dominant FAP and cyanobacterial populations in these mats (Klatt *et al*., in preparation). The metabolic potential of the SRB guild was based on several well-studied organisms (*Desulfovibrio vulgaris *Hildenborough, *Desulfotalea psychrophila*, *Desulfovibrio desulfuricans *G20, *Desulfobacterium *sp., and *Archaeoglobus fulgidus*), as well as *Thermodesulfovibrio yellowstonii*, a thermophilic SRB related to isolates from Mushroom Spring [6,60-63]. GenBank accession numbers for these genomes, in the stated order, are AE017285.1, CR522870.1, CP000112.1, CP001087.1, AE000782.1, and CP001147.1.

The use of guilds to minimize community complexity is useful for the compartmentalized approach. For the current study, guilds were defined based on four important parameters: energy sources, carbon sources, electron donors and electron acceptors. Each guild utilized a different combination of metabolites to satisfy these parameters. Subdominant guilds were not included in the model (*e.g*. methanogenic archaea). Here it is assumed that the methanogens would be responsible for degrading phototroph biomass buried within the mat. Since the control volume only considers the active phototrophic region, the decaying biomass would occur outside this control volume removing any possible role played by this subdominant guild.

The metabolic model input consists of the carbon and electron balanced substrates and products for each considered reaction. It should be noted that these organisms are not characterized at a level which would justify genome-scale reproductions. While such models are admirable for well documented organisms, problems with inaccurate, automated genome annotations call into question the benefit of such attempts with any but the best studied microbes [64]. The biomass compositions of these functional guilds have not been experimentally determined due to tight physical coupling of the cells within the microbial mats. The functional guilds reside within a distance of 100 μm. Biomass reactions were written to represent bacterial biosynthetic requirements for a composition of 78% proteins, 16% nucleic acids, and 10% other macromolecules. While the biomass compositions of actual mat constituents were not measured, previous published accounts [*e.g*. [48]] as well as unpublished observations from the authors suggest that overall yields are relatively insensitive to the biomass equation as long as the biomass has a biologically relevant degree of reduction (*e.g*. approximately 4.8 on an N_2 _basis). The supplemental material contains a degree of reduction analysis of the utilized biomass expression. Its degree of reduction is 4.7. The methods developed here could be applied with alternative biomass synthesis reactions based on future experimental knowledge. Carbon storage consisting of polyglucose or polyhydroxybutyrate was considered separately from biomass production. The metabolic models used in this study are included in additional file 1: Supplement.

Separate models were constructed to represent two distinct phases of a diel cycle, day and night. Differences between the modeled phases were based on the extensive geo- and biochemical data at these sites [5,11,12,55,65] and included sources and shuttles for carbon and energy as well as oxygen and light availability (see Figure 1 for graphical summary). The day and night metabolisms are connected through the fluxes of reduced carbon and fixed nitrogen. The amount of CO_2 _fixed during the day determines the mat's overall dark phase reduced carbon budget while the nitrogen fixed during the night using energy stored as polyglucose sets the consortium's fixed nitrogen budget.

Briefly, during the day, the *Synechococcus *spp. convert solar energy, CO_2_, and water into oxygen, ATP, and reduced carbon. Concurrently, the anoxygenic FAP derive energy from sunlight and exploit the reduced carbon (glycolate and acetate) produced by the *Synechococcus *guild. By-products of cyanobacterial activity also serve as carbon and energy sources for SRB. These interactions were used to guide selection of relevant organismal modes for the day nested approach.

At night, the mat was treated as anoxic due to the lack of oxygenic photosynthesis and low oxygen solubility at 60°C. This is a simplification: low levels of oxygen are actually available at the mat surface while most of the mat is anaerobic. The model *Synechococcus *guild ferments stored polyglucose into a variety of organic compounds including lactate, propionate, acetate, and formate as well as ethanol. In addition to being the primary producer of reduced carbon, *Synechococcus *is also thought to be the primary producer of fixed nitrogen for the mat [11]. These were the interactions used to guide first-round selection of modes for the night nested method. The nitrogen fixation reactions are considered solely in the night model because of the sensitivity of nitrogenase to oxygen. This could be a simplification of actual mat behavior: mRNA expression evidence and *in situ *activity assays suggest that a large fraction of nitrogenase synthesis occurs in the morning, when light-derived reducing equivalents are available but before the mat becomes oxic [5].

The Mushroom and Octopus Spring phototrophic mat communities are found within flowing geothermal springs. Consequently, it was assumed that the mass of each metabolite lost from the control volume due to convective transport was exactly equal to that gained by the control volume through convective transport from upstream microbes. The gases CO_2_, O_2 _and H_2 _were not constrained by this treatment and could vent to the atmosphere.

The nested models require the defining of 'transfer metabolites', which are permitted to serve as metabolic sinks and sources for the individual guild simulations, but not for the community simulation. They were chosen based on knowledge of the springs and compounds commonly transported across bacterial membranes and are shown in Figure 1, associated with arrows between guilds. Individual guild networks were then evaluated for elementary modes with 'transfer metabolites' allowed to accumulate (or deplete). This was accomplished by defining them as external metabolites in CellNetAnalyzer. The resulting modes were sorted in Excel based on product synthesis yields considered relevant to the system. These criteria are listed in additional file 1: Supplement. The modes with high yields, which represent overall guild stoichiometries, were then used as inputs for the second round of EMA (with 'transfer metabolites' now treated as internal metabolites in CellNetAnalyzer). The first round of EMA used enzyme substrate and product stoichiometries to determine how the numerous enzymes can work together as a system, while the second round of EMA used guild stoichiometries to determine how the guilds could work together as a system.

### Elementary mode analysis

Algorithms for calculating elementary modes from metabolic network topology are described elsewhere [46]. These algorithms are based on a field of mathematics known as convex analysis. Calculation of elementary modes in this work was performed using the software CellNetAnalyzer 9.0 and MATLAB v 7.6 [66]. CellNetAnalyzer is freely available to academics at http://www.mpi-magdeburg.mpg.de/projects/cna/cna.html, but requires MATLAB version 6.1 or higher. Due to computational burden, the models had to be constructed to avoid missing essential enzymatic activity while still permitting a computationally tractable network. Necessary simplifications included rounding, neglecting the accumulation of fermentation products at night, combining macromolecular syntheses into 'lumped' reactions based on draws from the central metabolism, using a proxy for cellular maintenance energy requirements, and streamlining treatment of nitrogen storage. The models presented in additional file 1: Supplement were used to produce all discussed results. A new algorithm for EMA has been recently described that permits the use of a 64 bit Linux based system [37]; this will likely permit analysis of larger networks than was previously possible. Algorithms for parallel processing have been proposed and will likely reduce the need for simplification, but are not yet publically available [67].

## Authors' contributions

DMW, RG, RPC, and WPI were the authors responsible for conception and design of the experiment. Data was acquired, analyzed, and interpreted by CGK, JEA, KB, NM, RPC, RT, SM, SM, and ZJ. The manuscript was drafted by CGK, JEA, KB, NM, RPC, RT, SM, SM, and ZJ. DMW, RG, RPC, and WPI revised the manuscript critically for intellectual content. All authors read and approved the final manuscript.

## Supplementary Material

Additional file 1 **Supplement**. This spreadsheet contains all additional information referenced in the text. This material includes the criteria used to select modes during the first round of processing for the nested models, a short illustration of the mechanisms used to separate metabolites by guild for the modeling methods, the script used to normalize first-round vectors for the nested approach, the full error results from the nested models, a sample calculation illustrating how modes were combined in the daytime case study, and the final models used to produce all discussed results (along with diagrams of the individual and pooled metabolic networks). Final network models will be submitted in SBML format to the BioModels database http://www.ebi.ac.uk/biomodels-main/ upon manuscript publication. Click here for file
